# Effects of Sequential Induction Combining Thermal Treatment with Ultrasound or High Hydrostatic Pressure on the Physicochemical and Mechanical Properties of Pea Protein–Psyllium Hydrogels as Elderberry Extract Carriers

**DOI:** 10.3390/ijms25169033

**Published:** 2024-08-20

**Authors:** Adonis Hilal, Anna Florowska, Tomasz Florowski, Katarzyna Rybak, Ewa Domian, Marcin Szymański, Małgorzata Wroniak

**Affiliations:** 1 Department of Food Technology and Assessment, Institute of Food Science, Warsaw University of Life Sciences, 02-787 Warsaw, Poland; anna_florowska@sggw.edu.pl (A.F.); tomasz_florowski@sggw.edu.pl (T.F.); malgorzata_wroniak@sggw.edu.pl (M.W.); 2 Department of Food Engineering and Process Management, Institute of Food Science, Warsaw University of Life Sciences, 02-787 Warsaw, Poland; katarzyna_rybak@sggw.edu.pl (K.R.); ewa_domian@sggw.edu.pl (E.D.)

**Keywords:** binary hydrogel, protein–polysaccharide interactions, delivery system, microstructure, textural properties, antioxidant properties, anthocyanins

## Abstract

Entrapping bioactive ingredients like elderberry extract in hydrogels improves their stability and functionality in food matrices. This study assessed the effect of sequential thermal treatment with ultrasound (US) or high hydrostatic pressure (HHP) and treatment duration on pea protein–psyllium hydrogels as elderberry extract carriers. Measurements included color parameters, extract entrapment efficiency, physical stability, textural properties, microrheology, FT-IR, thermal degradation (TGA), SEM images, total polyphenols content, antioxidant activity, and reducing power. The control hydrogel was obtained using only thermal induction. Both treatments impacted physical stability by affecting biopolymer aggregate structures. Thermal and US combined induction resulted in hydrogels with noticeable color changes and reduced entrapment efficiency. Conversely, thermal and HHP-combined induction, especially with extended secondary treatment (10 min), enhanced hydrogel strength, uniformity, and extract entrapment efficiency (EE = 33% for P10). FT-IR and TGA indicated no chemical structural alterations post-treatment. Sequential thermal and HHP induction preserved polyphenol content, antioxidant activity (ABTS = 5.8 mg TE/g d.m.; DPPH = 11.1 mg TE/g d.m.), and reducing power (RP = 1.08 mg TE/g d.m.) due to the dense hydrogel structure effectively enclosing the elderberry extract. Sequential thermal and HHP induction was more effective in developing pea protein–psyllium hydrogels for elderberry extract entrapment.

## 1. Introduction

In recent years, the growing consumer focus on health and wellness has significantly boosted the demand for functional foods, which are designed to provide additional health benefits beyond basic nutrition. These foods often incorporate bioactive ingredients such as essential oils, vitamins, prebiotics, and probiotics [1,2]. Lately, there has been an increasing trend in developing pro-healthy foods (functional foods) through the addition of natural extracts containing antioxidants. Among these, anthocyanins, including cyanidin-3-glucoside, cyanidin-3-sambubioside, and peonidin-3-glucoside, predominantly found in elderberry (*Sambucus nigra* L.), are recognized for their antioxidant properties [3,4]. Despite these advantages, anthocyanins are highly prone to degradation during food processing and storage, resulting in decreased bioavailability and effectiveness. Consequently, there is a critical need for effective strategies to stabilize and deliver these compounds in food systems [5,6,7].

Hydrogels, three-dimensional networks of cross-linked polymers, offer an innovative approach for the entrapment and protection of anthocyanins. Their unique characteristics, including softness, elasticity, and high water retention capacity, render them ideal matrices for enhancing the stability and delivery of sensitive ingredients [8,9,10]. Integrating hydrogels into food formulations can enable precise control over texture and mouthfeel, aligning sensory attributes with consumer preferences, while also serving as carriers for anthocyanins [10,11,12,13]. Selecting appropriate components for the hydrogel is crucial to achieving these desired functional and mechanical properties [14].

Combining two biopolymers can yield hydrogels with more customizable properties. Prior research indicates that pea protein and psyllium can serve as effective building blocks for a binary hydrogel matrix [14,15]. Pea protein’s gelation properties facilitate the formation of robust networks, which provide the hydrogel with necessary structural integrity [16]. This characteristic imparts mechanical strength and stability, critical for the hydrogel’s role as a carrier of anthocyanins. Studies have demonstrated that protein complexation, via direct binding and co-assembly, is an efficient method to enhance anthocyanin stability [17,18]. Psyllium, rich in the polysaccharide arabinoxylan, possesses notable water-binding capacity and gel-forming abilities, alongside its prebiotic benefits [19,20]. The incorporation of psyllium into pea protein-based hydrogels significantly enhances the hydrogel’s viscosity and stability, thereby augmenting its efficacy as a structural matrix and delivery system for anthocyanin entrapment [21,22,23]. While protein–psyllium hydrogels exhibit significant advantages, such as edibility, biocompatibility, improved functional properties, and notable nutritional benefits (including psyllium’s prebiotic properties), there are also notable challenges that need to be addressed. These include potential complexities in gelation control and sensory attributes that may affect consumer acceptance. These challenges require precise control through the formulation and processing conditions to achieve the desired gel properties [24,25].

Hydrogel induction refers to the processes employed to initiate and control the formation and structuring of hydrogels. Conventional methods for inducing gelation in pea protein–psyllium hydrogels typically rely on thermal induction, wherein heating is used to trigger the gelation [16,26]. However, this approach can destabilize sensitive bioactive compounds such as anthocyanins [5]. To overcome this limitation, a novel combined approach can be adopted. Initially, thermal induction is employed to induce protein gelation by heating the pea protein to its gelation temperature, thereby unfolding and forming an initial network. After cooling, psyllium and elderberry extract are incorporated, and the network formation is further refined by the assistance of high hydrostatic pressure (HHP) or ultrasound (US). These additional treatments enhance the structuring of the hydrogel network, improving its functional properties and stability, which enhances the entrapment of anthocyanins in the matrix [24,27]. The use of HHP (100 to 600 MPa) as an additional step in the induction enhances the network by promoting protein interactions through their further unfolding and aggregation [28,29]. Additionally, the physical compression caused by high pressure on the forming structure can influence the further entrapment of anthocyanins, leading to a dense and cohesive hydrogel. Meanwhile, US treatment improves the dispersion of biopolymers, resulting in a more homogeneous and organized hydrogel, which can also influence the anthocyanins entrapment efficiency [30]. Ultrasound treatment (30–100% amplitude) can be deployed in the restructuring of pea protein, which can affect its hydration properties, leading to a higher structural strength when compared to traditionally induced hydrogel [31]. This combined method aims to leverage the initial thermal gelation with the advanced structuring capabilities of HHP and US to develop new and highly customizable hydrogels. This combined method aims to leverage the initial thermal gelation with the advanced structuring capabilities of HHP and US to develop pea protein–psyllium hydrogel for anthocyanins entrapment.

Despite extensive studies exploring various protein–polysaccharide combinations for hydrogel formation, such as those involving animal-based proteins like whey and casein [32] and plant-based proteins like soy and pea [18,33], there is a notable gap in research concerning the specific synergy between pea protein and psyllium, particularly for anthocyanin-rich elderberry extract entrapment. Additionally, the potential of combining traditional thermal induction with advanced non-thermal methods like HHP and US remains underexplored, specifically in the context of influencing the physicochemical and mechanical properties of the matrices, as well as the entrapment efficiency of the elderberry extract. These gaps underline the necessity of investigating the unique combination of pea protein and psyllium, as well as the innovative application of sequential thermal and non-thermal induction techniques. This study hypothesizes that combining thermal induction with high hydrostatic pressure (HHP) or ultrasound (US) treatments will enhance the structuring of pea protein–psyllium hydrogels, thereby improving their functional properties, stability, and entrapment efficiency of the elderberry extract. The aim was to investigate these combined methods’ effects on the physicochemical and mechanical properties of the hydrogels, ultimately developing structures with improved integrity, stability, and enhanced antioxidant activity. By addressing these research gaps, this study contributes to the enhancement of functional foods and the development of innovative hydrogel-based delivery systems.

## 2. Results and Discussion

The analysis of color parameters (Table 1) reveals distinct effects of 5 and 10 min of ultrasound (U5 and U10, respectively) and high hydrostatic pressure (P5 and P10, respectively) treatments on the optical properties of hydrogels. The lightness (L*) remained consistent across all treatments compared to the control hydrogel (C), indicating no significant impact on brightness. However, ultrasound treatments (U5 and U10) significantly increased the a* values, indicating a shift towards the red spectrum, while high-pressure treatments (P5 and P10) significantly reduced a* values. For the b* values, ultrasound treatments resulted in a significant increase, whereas high-pressure treatments decreased this value. The chroma (C*) values were significantly increased by ultrasound treatments (U5 and U10), suggesting more saturated colors compared to the control hydrogel (C). Conversely, high-pressure treatments (P5 and P10) significantly decreased chroma compared to the control. The hue angle (h) showed a significant decrease in ultrasound-treated samples (U5 and U10), indicating a perceptible shift in hue towards the red spectrum. In contrast, high-pressure treatments (P5 and P10) caused a significant increase in hue angle compared to the control, indicating a shift towards the blue-green spectrum. These differences in a*, b*, chroma (C*), and hue (h) could be attributed to the formed structure and whether the extract is effectively distributed within it. When the extract became entrapped within a hydrogel structure, the environment surrounding the pigments changed, which might have impacted the hydrogels’ optical properties, reducing their ability to interact with light as freely as they would in a solvent [34]. To comprehensively determine the impact of the treatment type and duration on the color of the obtained hydrogels (U5, U10, P5, and P10) in comparison to the control hydrogel (C), the total color difference (ΔE) was determined (Table 1). Analyzing the obtained results, it was found that ΔE for U5 and U10 differed the most from the control sample. However, this color difference was only noticeable for experienced observers (1 < ΔE < 2). On the other hand, in the case of P5 and P10, the color difference was not noticeable for any observer (0 < ΔE< 1).

The analysis of extract entrapment efficiency (EE) reveals significant differences in the efficacy of ultrasound (U5 and U10) and high hydrostatic pressure (P5 and P10) treatments in entrapping extracts within the hydrogel matrix. Based on the results (Table 1), it was observed that ultrasound treatments (U5 and U10) significantly decreased the EE compared to the control, with U5 showing the lowest entrapment efficiency at 3% and U10 slightly higher at 9%. This could be due to the formation of a weaker gel structure, which was not able to entrap the extract. Subjecting biopolymers to the effect of ultrasound (US) treatment during induction leads to a cavitation process, which might slow down the formation of large aggregates. However, higher ultrasound treatment power or longer treatment duration has been proven to positively affect the technological and physical properties of biopolymers. The prolonged exposure to ultrasound (10 min) can lead to more uniformly dispersed aggregates (due to thinning effect), which improves the homogeneity of the gel structure [35,36]. This improved homogeneity can enhance the entrapment of anthocyanins by providing a more consistent network, which might explain the higher EE value compared to the shorter treatment duration (5 min). In contrast, high-pressure treatments (P5 and P10) significantly increased the EE compared to the control, with P5 achieving 20% and P10 reaching the highest entrapment efficiency at 33%. The increased EE observed in high-pressure-treated samples suggests that the application of high hydrostatic pressure enhances the structural integrity of the hydrogel matrix, facilitating the entrapment of anthocyanins. Similar tendencies were observed in the research of Mao et al. [37] focusing on the effect of HHP on riboflavin-loaded soy protein isolate cold gel. This increase in the entrapment efficiency of the extract can be attributed to the sequential thermal and high hydrostatic pressure (HHP) induction. This process enhanced the swelling of the gel network, thereby improving the retention of the extract within the hydrogel matrix [38].

The observed differences in the entrapment efficiency (EE) of elderberry extract between ultrasound-induced and high-pressure-induced hydrogels explain the variations in a*, b*, chroma (C*), and hue (h) values (Table 1). High-pressure treatments compressed the hydrogel matrix, creating a dense network that effectively entrapped the extract. This resulted in reduced a*, b*, and C* values, with the extract distributed and entrapped within the structure, shifting the hue (h) towards the blue-green spectrum. Conversely, ultrasound treatments produced a weaker hydrogel structure, leading to low extract entrapment, increasing a*, b*, and C* values. The extract was more concentrated near the surface, shifting the hue (h) towards the red spectrum.

**Figure 1:**
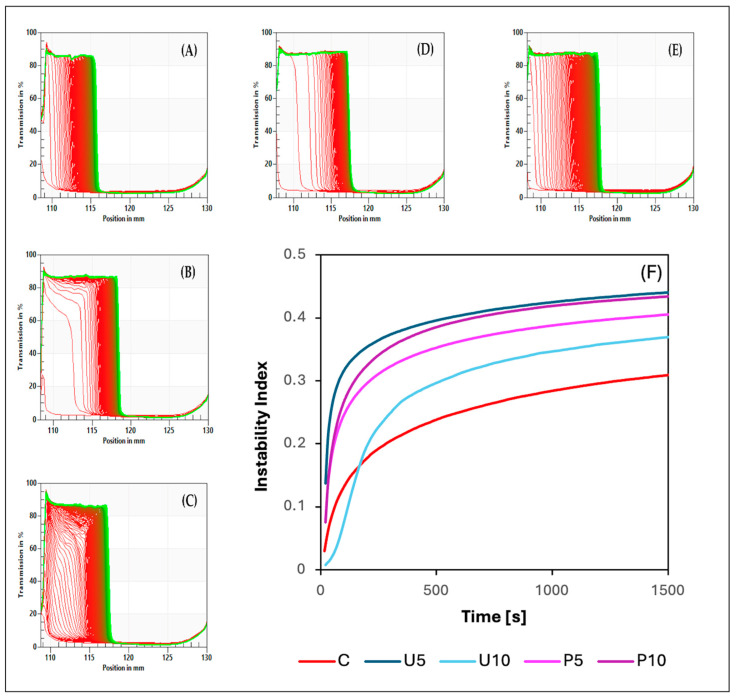
Effects of sequential thermal and non-thermal induction on the transmission profiles “fingerprints” (**A**–**E**) and the destabilization kinetics (**F**) of the obtained hydrogels (*n* = 3). Samples description: (**A**) control hydrogel C induced via thermal induction; (**B**,**C**) thermal induction followed by ultrasound treatment for 5 and 10 min, U5 and U10, respectively; (**D**,**E**) thermal induction followed by high hydrostatic pressure treatment for 5 and 10 min, P5 and P10, respectively. For (**A**–**E**), the red lines represent light extinction at the starting point, and the green lines represent light extinction at the end of the analysis.

STEP technology, which stands for space-time-resolved extinction profiles, provides distinct transmission profiles that act as unique “fingerprints” (Figure 1A–E), reflecting fluctuations in particle concentration within analyzed samples [39]. These profiles are crucial for assessing the kinetics of particle concentration changes induced by centrifugal forces, resulting in phase separation within the samples. By studying these transmission profiles, observable structural compression occurred, evident in decreased light transmission in the right quadrant of each graph. The propagation of destabilization within the samples moved downward, indicating maximal particle concentration towards the base of the sample, while a less particle-concentrated liquid phase was observed in the upper regions. Based on the destabilization kinetics (Figure 1F), it can be concluded that destabilization in U5, as well as P5 and P10, occurred the quickest, followed by U10. Conversely, the control sample (C) exhibited the slowest destabilization changes. Subjecting the samples to ultrasound (US) and high hydrostatic pressure (HHP) treatment might disrupt weaker interactions that could have formed between the biopolymers, leading to a less physically stable structure. Additionally, these treatments might affect protein stability from a thermodynamic perspective, decreasing the kinetic stability and increasing the unfolding rate. Folded proteins can act as active fillers in a hydrogel structure; thus, the complete unfolding of all proteins might decrease mechanical rigidity, thereby affecting network formation and relaxation [24,40].

The physical stability of the obtained hydrogels was assessed using the instability index, which ranges from 0 (indicating stability) to 1 (indicating instability). Table 2 presents the average instability index values for each sample. The control hydrogel (C) exhibited the highest physical stability. Both ultrasound (US) and high hydrostatic pressure (HHP) treatments, along with increased treatment durations, led to a decrease in physical stability. This reduction in stability is likely attributed to the impact of these treatments on the size and density of pea protein–psyllium aggregates, which may have resulted in a more heterogeneous microstructure and a diminished water-holding capacity in the hydrogels [39,41].

The data presented in Table 2 reveal significant impacts of ultrasound and high-pressure treatments on the textural and microrheological parameters of hydrogels. Ultrasound treatments generally decreased strength, adhesion, spreadability, and elasticity while increasing the solid–liquid balance, suggesting a disruption of the hydrogel network. This effect is attributed to cavitation forces that disrupt protein–polysaccharide aggregates, leading to a drop in the structural elasticity (EI). Both the application of US and its duration influenced the viscoelastic properties of the samples, with elastic properties still being predominant (SLB < 0.5). This aligns with the available literature, suggesting the increase in liquid phase movement between the structures caused by the formation of a weak network [42,43]. Conversely, high-pressure treatments increased strength and spreadability while also increasing the solid–liquid balance (SLB). P10 exhibited the highest values of textural properties and SLB (which was still below 0.5), indicating a gel structure formation capable of entrapping the extract-rich liquid phase (EE = 33%). Similar observations were made by Luo et al. [28] in their investigation of HHP’s impact on quinoa protein gelation, suggesting that HHP induces the formation of a heterogeneous structure with large protein aggregates. The heterogeneity in the formed gel structure for P5 and P10 might have played a crucial role in contributing to structural collapse.

**Figure 2:**
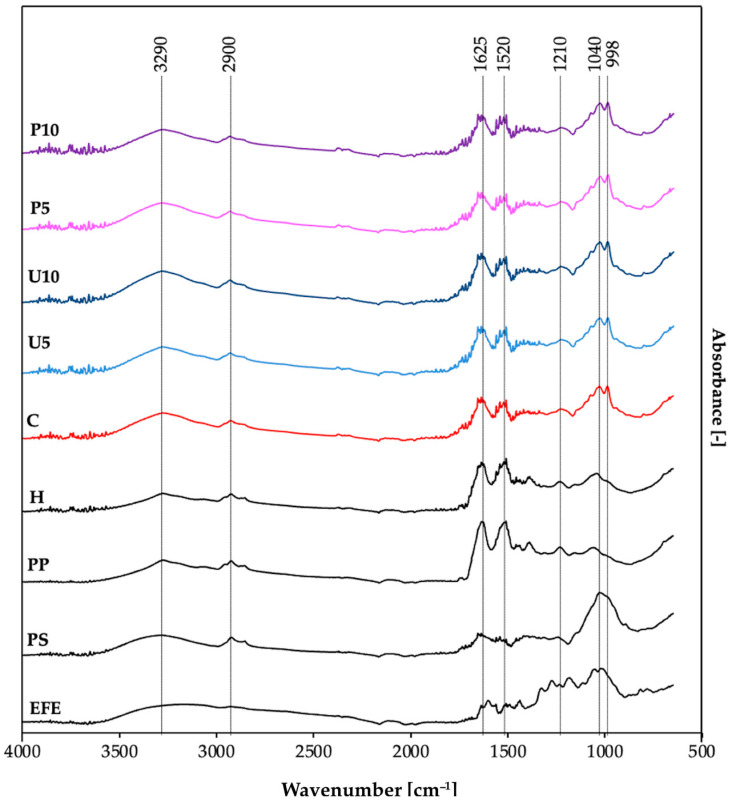
FT-IR spectra for the building blocks (EFE, PP, and PS) and the obtained hydrogels (H, C, U5, U10, P5, and P10). Samples description: EFE—Elderberry fruit extract; PS—Psyllium; PP—Pea protein; H—Pea protein–psyllium hydrogel without elderberry fruit extract; C—Control hydrogel induced via thermal induction; U5 and U10—Thermal induction followed by ultrasound treatment for 5 and 10 min, respectively; P5 and P10—Thermal induction followed by high hydrostatic pressure treatment for 5 and 10 min, respectively.

The physicochemical and mechanical properties of the obtained matrices, including mechanical characteristics, structural morphology, and entrapment efficiency, are influenced by intermolecular forces among the building blocks. Fourier transform infrared spectroscopy (FT-IR) was employed to analyze these interactions by identifying molecular vibrations and stretching patterns of specific molecular groups within the samples (Figure 2). FT-IR analysis was conducted individually for each building block—elderberry fruit extract (EFE), pea protein (PP), and psyllium (PS). Subsequently, the analysis was conducted collectively for all prepared hydrogels (H, C, U5, U10, P5, P10), where H was a pea protein–psyllium hydrogel without elderberry fruit extract. The obtained FT-IR spectra revealed four main regions: the “single bond region” (2500–4000 cm^−1^), the “triple bond region” (2000–2500 cm^−1^), the “double bond region” (1500–2000 cm^−1^), and the “fingerprint region” (1500–600 cm^−1^), which are unique to specific molecules. All analyzed samples exhibited distinct peaks, affirming their organic origin [44].

The FT-IR spectra analysis of the elderberry fruit dry extract (EFE) revealed six major peaks. Given that the extract contains 25% anthocyanins, the overlapping of characteristic peaks of anthocyanins and other compounds (such as phenolic compounds) was considered. The peak observed in the region between 1600 and 1650 cm^−1^ represents the stretching vibrations within the aromatic rings present in the anthocyanidin core [45]. The peaks in the region of 1000–1250 cm^−1^ represent C-O stretching in aromatic rings and C-O-C glycosidic linkages, indicating that sugars (such as sambubiose) are attached to the anthocyanidin core. The broad band in the region 3300–3500 cm^−1^ indicates the presence of hydroxyl groups (O-H) [46].

Psyllium husk (PS) polysaccharide is composed of β-1,4-linked D-xylose units (xylan) and arabinoxylan (a β-1,4-linked D-xylose backbone with α-1,3-linked L-arabinose side chains). Based on the FT-IR spectra (Figure 2) and previous research by Waleed et al. [47], the following characteristic peaks can be observed: the polysaccharide backbone region (1000–1250 cm^−1^), which encompasses various stretching vibrations such as C-O-C stretching between sugar units in the xylan and arabinoxylan backbone, and C-O stretching arising from the hydroxyl groups. The small peak at approximately 1630 cm^−1^ is associated with the C=O stretching vibration of acetyl groups present in some xylan and arabinoxylan molecules. The intensity of this peak correlates with the degree of acetylation, indicating that the psyllium used had a low degree of acetylation. This low acetyl group content leads to a higher affinity for water and increased final viscosity. The peak around 3300–3500 cm^−1^ suggests the presence of O-H stretching of hydroxyl groups on the sugar ring and may also indicate the presence of intermolecular hydrogen bonding [48].

The pea protein (PP) exhibited a characteristic peak in the Amide I band region (1600–1700 cm^−1^) associated with C=O stretching vibrations, which are the strongest and correlate with the protein’s secondary structure. The peak position at 1625 cm^−1^ suggests that the dominant secondary structure is β-sheet. Additionally, the Amide II band (1550 cm^−1^), associated with N-H bending and C-N stretching vibrations, may have been overlapped by the characteristic C-C stretching vibrations of aromatic amino acid side chains (1500–1600 cm^−1^), indicating the presence of phenylalanine and tyrosine in the pea protein [49]. The peak at 2900 cm^−1^ strongly indicates C-H stretching vibrations in the aliphatic side chains of amino acids such as alanine, valine, leucine, isoleucine, and proline. The peak at 3290 cm^−1^ represents N-H stretching vibrations from the primary amine (NH_2_) groups present in the amino acid backbone of pea protein [50].

The hydrogel obtained from pea protein and psyllium husk without the addition of EFE (H) exhibited new interactions between these molecules. The disappearance of the strong peak between 1000 and 1200 cm^−1^ in the H hydrogel spectrum, compared to the reference spectrum of pure psyllium husk (PS), indicates interactions between the hydrogel components likely due to hydrogen bond formation between the sugar units and protein. Additionally, a decrease in the intensity of the peaks between 1400 and 1700 cm^−1^ suggests structural changes in the pea protein, including unfolding and aggregation. The broadening of these peaks may reflect the presence of a mixture of different secondary structures caused by heating [49]. In contrast, Niu et al. [21] observed increased intensity of absorption peaks at 1401 and 1648 cm^−1^ in psyllium and whey protein interactions, indicating the formation of additional C=O and C-N bonds likely resulting from the carbonyl-ammonia reaction.

Obtaining a pea protein–psyllium hydrogel with the addition of elderberry fruit extract (EFE) caused significant changes in the presented spectrum (C). A new overlapping double peak (850 and 1200 cm^−1^) was observed, likely indicating hydrogen bonding between hydroxyl groups (OH) of anthocyanins (present in the elderberry extract, EFE) and carbonyl groups (C=O) of pea protein, resulting in a modified vibrational pattern. Additionally, a slight increase in the peak at ~3300 cm^−1^ might be attributed to the presence of additional hydroxyl groups (OH) from the added extract. The broadness of this peak suggests that the OH groups exist in different chemical environments within the sample—some free or weakly interacting, while others are involved in hydrogen bonding with functional groups in pea protein or psyllium.

Sequential thermal, ultrasound (U5, U10), and high hydrostatic pressure (P5, P10) induction did not significantly alter the FT-IR spectra. These findings suggest that ultrasound (US) and high hydrostatic pressure (HHP) affect the overall morphology—such as pore size, density, and homogeneity—without significantly modifying the chemical bonds within the hydrogel matrix. This observation aligns with the study by Jambrak et al. [51], where ultrasound treatment on soy protein isolate revealed minimal changes in chemical composition based on FT-IR spectra. However, alterations in protein aggregation behavior were noted, indicating changes in protein interactions without affecting individual amino acid bonds. Similarly, ultrasonic treatment of chitosan exhibited no significant changes in characteristic peaks, suggesting the primary chemical structure remained intact while changes in crystallinity were observed [52]. Conversely, some studies report that US treatment can alter the conformation and chemical interactions of biopolymers due to cavitation-induced temperature increases, leading to protein denaturation and degradation of thermolabile components [53]. In our study, US treatment was performed in an ice bath to mitigate overheating, and the absence of changes in FT-IR spectra may be attributed to the pre-denatured state of pea proteins from prior thermal induction. In the case of HHP treatment, previous studies on soy protein and flaxseed gum indicated a decrease in glycation sites and alterations in protein secondary structure [54]. Jin et al. [55] demonstrated that HHP could induce morphological changes in soy protein hydrolysates/β-glucan/ferulic acid complexes, transforming them from primarily spherical to irregular shapes, influenced predominantly by binding interactions among the components. Moreover, HHP has been shown to significantly modify molecular weight distribution, average particle size, and morphological arrangement or orientation of various polysaccharides [56].

**Figure 3:**
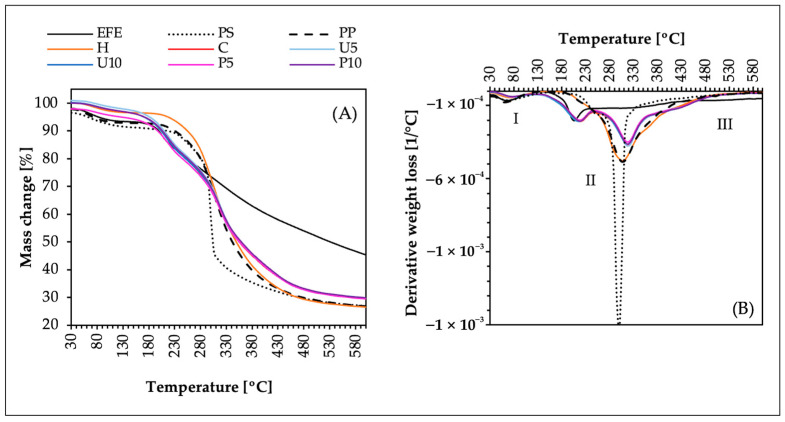
Phases of the thermal degradation analysis of the building blocks (EFE, PP, and PS) and the obtained hydrogels (H, C, U5, U10, P5, and P10). (**A**) Thermogravimetric analysis (TGA) and (**B**) derivative thermogravimetry (DTG). Samples description: EFE—Elderberry fruit extract; PS—Psyllium; PP—Pea protein; H—Pea protein–psyllium hydrogel without elderberry fruit extract; C—Control hydrogel induced via thermal induction; U5 and U10—Thermal induction followed by ultrasound treatment for 5 and 10 min, respectively; P5 and P10—Thermal induction followed by high hydrostatic pressure treatment for 5 and 10 min, respectively.

To gain a more comprehensive understanding of interactions and potential changes within the hydrogels, the thermogravimetric analysis (TGA and DTG) of the obtained samples was performed. By analyzing both the functional group interactions (FT-IR) and the thermal behavior (TGA and DTG), it is possible to indicate if the occurring interactions between the components potentially influence the overall thermal stability. Figure 3 shows TGA (A) and DTG (B) of the analyzed samples. All samples showed an initial phase (I), between 30 °C and 130 °C, related to the evaporation of moisture or adsorbed water from the samples, as well as the loss of volatile compounds. The dried samples contained a low amount of water, resulting in minimal mass losses ranging from 2.8% to 8.5%—the highest mass loss was in the case of psyllium (PS). The main decomposition phase (II) was observed between 130 °C and 480 °C and can be divided into two sub-phases. The mass loss in this phase ranged from 39% in the case of EFE to 67% in the case of H (pea protein–psyllium hydrogel without elderberry fruit extract). The lower sub-phase (130–240 °C) represents the start of the thermal degradation of low-stability components, including side chains and low-molecular-weight oligomers. In this sub-phase, a weight loss was observed only in the case of elderberry fruit extract (EFE) and the hydrogels containing the extract (U5, U10, P5, and P10). This substantial weight loss corresponds to the degradation of sensitive compounds like anthocyanins, other polyphenols, and vitamins [57]. The incorporation of elderberry extract into pea protein–psyllium hydrogels (no matter the induction technique) resulted in a slight increase in the thermal resistance to degradation from 209 °C to 220 °C. The opposite observation was made by Cetinkaya et al. [58] in their study on gelatin nanofibers with black elderberry. They observed a slight decrease in the thermal resistance on the sample after mixing gelatin and the elderberry extract together (from 212 °C to 190 °C). The upper sub-phase of the main decomposition phase (II) occurred between 240 °C and 480 °C. In this sub-phase, a thermal degradation was observed in the case of all the analyzed samples except for the elderberry extract (EFE). Furthermore, the samples containing EFE (U5, U10, P5, and P10) showed slightly smaller weight loss (~64%) than pea protein–psyllium hydrogel without elderberry fruit extract (H) ~67%. During this sub-phase the most significant weight loss was observed, which is due to the primary structures breaking down into smaller, volatile molecules, often resulting in the release of CO, CO_2_, H_2_O, and various hydrocarbons [59]. In the third phase (III), the temperature range was from 480 °C to 600 °C. The weight loss during this last phase was similar to all the analyzed samples ~3% except EFE, for which the weight loss was approximately 9%. This is due to elderberry extract (EFE) containing more thermally labile organic compounds that decompose and volatilize over a wide temperature range. Thus, it can be summarized that the samples containing elderberry extract (U5, U10, P5, and P10) were more stable than elderberry extract by itself (EFE), which might be due to the formed hydrogel structure and the polyphenols–protein interactions. These hydrogels have similar degradation phases to those of their building components (PP and PS). Sequential thermal-, ultrasound (U5, U10)-, and high hydrostatic pressure (P5, P10)-induced hydrogels showed similar thermal stability in all phases compared to control hydrogel (C), indicating that these additional treatments did not significantly alter the thermal degradation profile of the samples.

**Figure 4:**
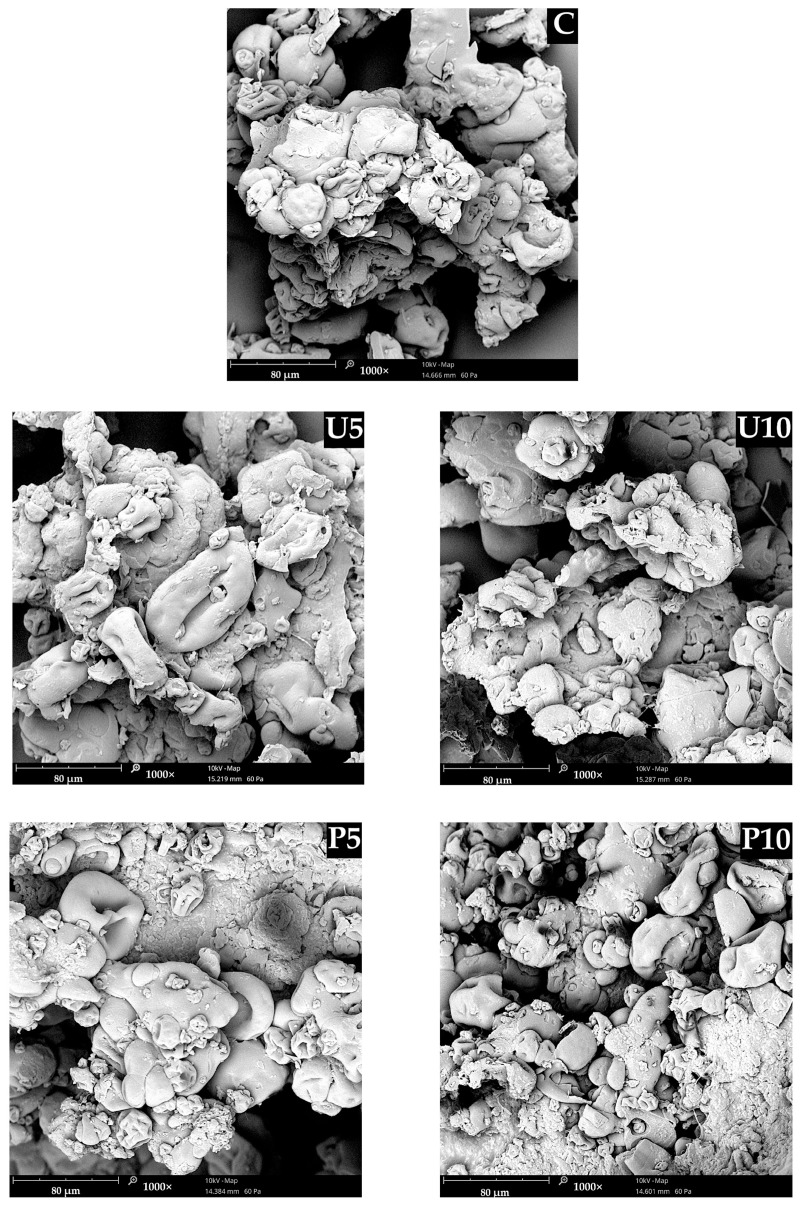
Effects of sequential thermal and non-thermal induction on the microstructure of the hydrogels. Magnification 1000×. Samples description: C—Control hydrogel induced via thermal induction; U5 and U10—Thermal induction followed by ultrasound treatment for 5 and 10 min, respectively; P5 and P10—Thermal induction followed by high hydrostatic pressure treatment for 5 and 10 min, respectively.

Based on the analysis of SEM microscopic images (Figure 4), it can be concluded that sequential thermal-, ultrasound (U5, U10)-, and high hydrostatic pressure (P5, P10)-inductions affected the microstructure of the obtained hydrogels. It was also noticed that the treatment duration also had an impact on the final structure. The microstructure of U5 appeared to have fewer fractured aggregate boundaries than U10. Additionally, the longer ultrasound treatment resulted in diminished variations in the size and distribution of the formed aggregates. This is due to the increased cavitation generated by the sound waves, creating rapid pressure fluctuations [60]. On the other hand, the microstructure of the hydrogels obtained via sequential thermal and high hydrostatic pressure treatment was characterized by a more compact structure when compared to U5, U10, and C. Out of all the analyzed samples, P10 had the most well-spanned microstructure, with fewer voids present between the aggregates; this explains the highest extract entrapment efficiency (EE) presented in Table 1. Multiple studies have reported consistent findings regarding the impact of high hydrostatic pressure (HHP) on biopolymeric hydrogels [28,61,62]. The sequential thermal and high hydrostatic pressure (HHP) induction could have enhanced the aggregation process, resulting in a compact and cohesive network that was able to physically confine the water phase containing the extract, while also increasing the binding sites between the pea protein, psyllium, and anthocyanins present in the extract. Moreover, this denser and more entangled hydrogel network minimized the voids in the structure, increasing the water-holding capacity and restricting the diffusion of the extract, thereby ensuring effective entrapment [63,64,65]. A similar relationship between structural density and entrapment efficiency was observed by Liu et al. [10] in their studies on gelatin–gellan gum hydrogels for the release of anthocyanins in the digestive system. They attributed this relationship to the water-soluble nature of anthocyanins present in the extract, which affected their entrapment due to the increase in water fixation within the gel network; this increased the entrapment of the extract.

Table 3 presents the effects of sequential thermal, ultrasound (U5 and U10), and high hydrostatic pressure (P5 and P10) treatments on the total polyphenol content (TPC) of pea protein–psyllium hydrogels. This parameter subsequently influences antioxidant activity and reducing power. The TPC for samples subjected to additional 5-min ultrasound (U5) or high hydrostatic pressure (P5) treatments did not show significant deviations compared to the control hydrogel (C). The TPC values ranged from 16,987 to 18,865 mg chlorogenic acid equivalents/100 g (d.m.) for U5 and P5, respectively. However, extending the treatment time to 10 min had different effects: for U10, the TPC decreased, while for P10, the TPC increased. The stability of polyphenols is influenced by various factors, including the presence of oxygen, pH levels, temperature, and other elements [66]. The extended ultrasound treatment (U10) resulted in polyphenol degradation. In contrast, the 10-min high HHP treatment (P10) enhanced polyphenol retention, correlating with increased extract entrapment efficiency (EE) as noted in Table 1. This improved entrapment likely protected the polyphenols from degradation. In the control hydrogel, with an EE of only 19%, the anthocyanins were inadequately protected, leading to lower TPC values compared to the P10-treated samples. Similar trends were observed in antioxidant activity and reducing power measurements. The ultrasound treatment (US) significantly reduced the free radical scavenging activity measured by the ABTS assay compared to the control hydrogel (C). On the other hand, U5 had higher values of DPPH and RP than C, which could be due to the increased homogeneity of the matrix, leading to a more uniform distribution of the extract in the hydrogel. Notably, increasing the duration of US treatment from 5 (U5) to 10 min (U10) decreased the values of the DPPH assay and reducing power (PR). This could be due to the formation of a weak gel structure unable to effectively entrap the extract, as well as degradation caused by localized high temperatures and oxidation from the cavitation effect, which introduced oxygen into the system [67,68]. On the contrary, increasing the HHP treatment time from 5 (P5) to 10 min (P10) caused a significant increase in all the assessed parameters. This improvement might be attributed to the formation of a more compact gel structure (Figure 2), which entrapped a larger amount of the elderberry fruit extract (EFE), protecting it from degradation. Additionally, HHP treatment likely caused air bubbles trapped in the system to escape to the hydrogel surface, reducing oxidation of the entrapped extract [62,69].

**Figure 5:**
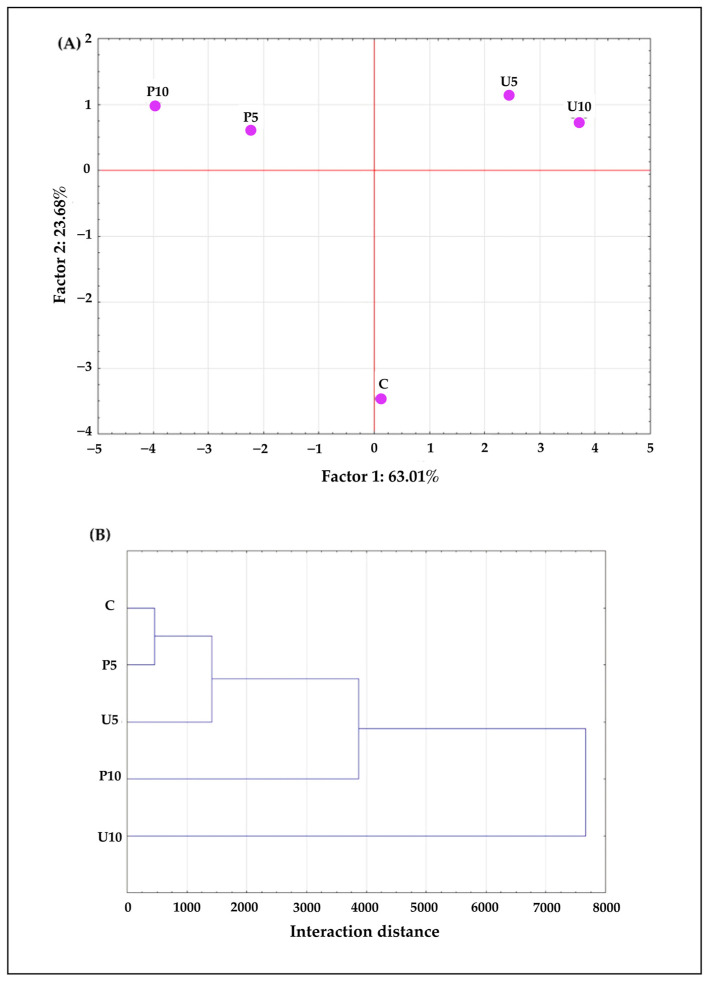
Principal component analysis PCA (**A**) and hierarchal cluster analysis HCA (**B**) of the obtained hydrogels.

Principal component analysis (PCA) and hierarchical cluster analysis (HCA) were applied to the obtained results to efficiently summarize the data collected in this study. PCA was performed using 16 active variables. Two principal components were identified (Figure 5A): component 1 (Factor 1) accounted for 63.01% of the variance, and component 2 (Factor 2) accounted for 23.68% of the variance. Together, these components explained 86.7% of the total variance in the results. Component 1 is strongly positively correlated with chroma C* (r = 0.96). On the other hand, a negative contribution of this factor was found for spreadability (r = −0.97), strength (r = −0.95), EE (r = −0.91), TPC (r= −0.88), ABTS (r = −0.98), DPPH (r = −0.74), and RP (r = −0.64). Component 2 is positively correlated with the SLB (r = 0.91) and instability index (r = 0.91). The negative contribution of factor 2 can be observed for EI (r = −0.89) and adhesion (r = −0.86). Given these interdependencies, the first principal component can be interpreted as a measure of the induction method that can attribute to the entrapment of elderberry extract and, consequently, the overall antioxidant activity. The second component can be interpreted as a measure of the induction method, contributing to the overall physical stability of the formed gel structure. Based on the sample distribution within the principal component space (Figure 5A) and the interaction distances observed in HCA (Figure 5B), it can be concluded that sequential thermal, ultrasound, and high hydrostatic pressure induction result in hydrogels with significantly different properties. The sequential thermal and ultrasound induction resulted in hydrogels with low structural stability and elasticity, negatively affecting the entrapment efficiency of the elderberry extract and the final antioxidant activity. Furthermore, extending the treatment time to 10 min (U10) may have caused additional degradation of the polyphenols, explaining the highest observed interaction distance (Figure 5B). On the other hand, the sequential thermal and high hydrostatic pressure induction resulted in hydrogels with high entrapment efficiency due to the formation of a compact gel structure capable of entrapping the extract. However, this structural compression also led to high SLB values. Nonetheless, these systems exhibited high antioxidant activity, with 10 min of HHP treatment yielding the highest total polyphenol content and antioxidant activity.

## 3. Materials and Methods

### 3.1. Materials

Pea protein (NUTRALYS^®^ F85F, protein content 88%, ash 10%) was obtained from Roquette Freres (Lestrem, France). Psyllium husk powder (PS, type 10351, purity: 95%, particle size: 60 mesh) was obtained from C.E. Roeper GmbH (Hamburg, Germany). Elderberry fruit dry extract (EFE, min. 95% pass 80 mesh, anthocyanins content 29%, polyphenols content 40%, carrier: maltodextrin) was obtained from GreenVit (Zambrów, Poland). Citric acid, sodium citrate, and NaCl were purchased from the local food ingredient supplier Agnex (Białystok, Poland).

### 3.2. Hydrogel Preparation

The hydrogel preparation process was based on previous studies with slight modifications [14,15]. It involved hydrating pea protein (12.5 g of protein/100 g of hydrogel) in distilled water for 60 min under constant stirring (300 rpm) using a heated magnetic stirrer. After that, the protein dispersion underwent heating at 80 °C for 30 min. Post-cooling to 20 °C, the pH of the dispersion was adjusted to 3 (using citric acid and sodium citrate), and the ionic strength was modified by adding NaCl (0.3 M). Psyllium husk (0.5 g/100 g of hydrogel) and elderberry dry extract (2 g/100 mL of hydrogel’s water phase) were introduced, and the dispersion was mixed for 10 min (300 rpm). The samples were divided into three groups (Table 4). One group was control hydrogels induced via thermal induction (C). Another group was thermal induction followed by ultrasound treatment (U) (25 kHz, 70 W, 100% pulse, 100% amplitude, sonotrode immersion at 15 mm) for 5 (U5) and 10 (U10) minutes using the ultrasound homogenizer P200St equipped with a titanium sonotrode S26d7 (Hielscher Ultrasonics GmbH, Teltow, Germany). The ultrasound treatment of the dispersions was conducted in an ice bath to prevent the samples from overheating (the temperature was kept at 20 ± 1 °C). The third group was thermal induction followed by high hydrostatic pressure treatment (P) (500 MPa, 20 °C) for 5 (P5) and 10 (P10) minutes using the U5000/120 Pascalizer (Unipress, Warsaw, Poland). Then, the obtained dispersions were stored for 24 h at 4 ± 1 °C to develop a gel structure. Once this duration elapsed, the samples were conditioned to a temperature of 20 ± 1 °C, following which they underwent testing to evaluate their properties. Table 4 explains the coding and inductions used for the hydrogels.

### 3.3. Color Parameters Measurements

A Minolta CR-5 colorimeter (Minolta, Japan; light source D65; measuring head hole: 8 mm) was used to measure the color components in the CIE L* a* b* system at the surface of the obtained hydrogels [14]. Through color measurement, four components were derived—L* (brightness), a*, b*, C* (chroma), and h (hue). The L*, a*, and b* components enabled the calculation of the color difference coefficient, expressed as delta E (ΔE). This coefficient quantifies the disparity between two colors and is determined by the following formula:

(1) $$\Delta E=\sqrt{{\left(L_{C}^{*}-L_{P}^{*}\right)}^{2}+{\left(a_{C}^{*}-a_{P}^{*}\right)}^{2}+{\left(b_{C}^{*}-b_{P}^{*}\right)}^{2}}$$

where $L_{C}^{*}$; $a_{C}^{*}$; $b_{C}^{*}$ refer to the color parameters of the control hydrogel (C) and $L_{P}^{*}$; $a_{P}^{*}$; $b_{P}^{*}$ refer to the color parameters of hydrogels induced with the additional treatment. The reported values represent the averages of three replicates. The extent of color difference between samples can be interpreted based on ΔE values: not perceptible to the observer (0 < ∆E < 1), detectable by experienced observers (1 < ∆E < 2), noticeable by unexperienced observers (2 < ∆E < 3.5), distinctly visible color difference (3.5 < ∆E < 5), and clear differentiation of two colors by the observer (5 < ∆E) [70].

### 3.4. Entrapment Efficiency (EE) Measurements

The entrapment efficiency was calculated based on the entrapped anthocyanins determined using the pH differential method with a spectrometer, based on the methodology of Ge et al. [71] with slight modifications. The samples were centrifuged for 10 min at 10,000 rpm. Subsequently, 20 mL of the resulting liquid phase was diluted to 100 mL with distilled water. Then, 5 mL of the prepared solution was further diluted to 25 mL using potassium chloride buffer (pH 1.0) and sodium acetate buffer (pH 4.5). The samples were stored in a dark place for 20 min at room temperature (20 ± 1 °C). Following this incubation period, the absorbance was measured at wavelengths of 510 and 700 nm against distilled water using a UV–VIS spectrophotometer (Genesys 180, ThermoScientific, Boston, MA, USA). The total anthocyanin content of the liquid phase was calculated using Equation (2) as follows:

(2) $$C=\frac{(A_{\mathrm{pH}1.0}-A_{\mathrm{pH}4.5})\times M_{w}\times DF\times 1000}{\varepsilon \times l}$$

where *A*_pH1.0_ and *A*_pH4.5_ are the maximum absorbance of the sample diluted with the buffers at pH 1.0 and 4.5, respectively; *M_w_* is the molecular weight of cyanidin-3-O-glucoside (449.2 g/mol); *DF* is the dilution factor; *ε* is the extinction coefficient (26,900 L/mol·cm); *l* is the path length (1 cm); and 1000 is the conversion factor from grams to milligrams. The anthocyanin entrapment efficiency was calculated using Equation (3) [72] as follows:

(3) $$\mathrm{EE}=\frac{C_{B}-C_{A}}{C_{B}}\times 100$$

where *C_B_* and *C_A_* are the total anthocyanin content of the sample before and after centrifugation, respectively. The analysis was performed in triplicate.

### 3.5. Physical Stability and Destabilization Behavior Measurements

The physical stability and destabilization kinetics of the hydrogels were evaluated using the LUMiSizer 6120-75 (L.U.M. GmbH, Berlin, Germany), which operates based on STEP technology (Space and Time Extinction Profiles), involving centrifugation under near-infrared (NIR) light [14,39]. For this analysis, the following parameters were used: dispersion volume of 1.8 mL, 870 nm wavelength, 1500 rpm, 15-h 10-min experiment duration, 210-s intervals, and temperature 20 °C. The transmitted light intensity was monitored over time and position across the sample length using SepView 6.0 software (L.U.M. GmbH, Berlin, Germany). The destabilization behavior (fingerprint) was derived from recorded data, and an instability index was calculated based on three replicates’ averaged values.

### 3.6. Textural Measurements

Textural analysis of the hydrogels was conducted using a TA.XT Plus texture analyzer (Stable Micro Mixtures, Surrey, UK) equipped with a 5 kg load cell and specific probes. A 0.5 cm diameter cylindrical flat probe (P/0.5R) measured hydrogel strength [N] and adhesion [N], with a set penetration depth of 8 mm, a measurement speed of 1.0 mm/s, and a temperature of 20 °C. Spreadability [N∙s] was assessed using a TTC Spreadability Rig at a measurement speed of 3.0 mm/s [39]. Data analysis was performed using Exponent version 6.1.4.0 software (Stable Micro Mixtures, Surrey, UK). Reported values represent averages from three replicates.

### 3.7. Microrheological Measurements

The microrheological properties of the hydrogels were studied using a Rheolaser Master device (Formulaction, L’Union, France) employing near-infrared light at 650 nm wavelength and the MS-DWS technique. Backscattered waves interference was captured by the detector, and Rheotest software 1.4.0.11 recorded the results. Parameters derived from raw data included mean square displacement (MSD) curves, elasticity index (EI) [nm^−2^], and solid–liquid balance (SLB) [-]. SLB is the ratio of elastic modulus (G′) to viscous modulus (G″). EI, calculated from the reciprocal of MSD at the plateau, indicates the proportionality to G′ [14,73]. Reported values are averages from three replicates.

### 3.8. Fourier Transform Infrared Spectroscopy Measurements

Infrared spectra were measured using a Cary 630 spectrophotometer (Agilent Technologies Inc., Santa Clara, CA, USA) equipped with a single-bounce attenuated total reflectance (ATR) diamond crystal interface. Before conducting FT-IR analysis, the hydrogels underwent freezing at −20 °C followed by freeze-drying. The resulting freeze-dried samples were ground into a powdered form. FT-IR measurements were performed within a wavelength range of 500–4000 cm^−1^, utilizing 32 scans at a resolution of 4 cm^−1^ [74]. Analysis involved pressing the dried sample against a crystal using a pressure clamp, with five scans recorded for each sample. Data collection was executed using MicroLab FTIR software 5.7.

### 3.9. Thermal Degradation Measurements (TGA and DTG)

Thermal stability was assessed using a thermogravimeter (TGA/DSC 3+, Mettler Toledo, Greifensee, Switzerland). Approximately 5 mg of the crushed material was placed in open 70 µL alumina crucibles and subjected to pyrolysis, ranging from 30 to 600 °C, with a heating rate of 10 °C per minute, under a nitrogen atmosphere (flow rate of 50 mL/min) [75]. The thermograms were analyzed using the STAR software (version 16.10) from Mettler Evaluation. To provide a comprehensive understanding of the thermal behavior, both TGA (thermogravimetric analysis) and DTG (derivative thermogravimetry) are presented. The analysis was performed in triplicate.

### 3.10. Microstructure Morphology—SEM Analysis

To examine the microstructure of the freeze-dried hydrogel samples, they were mounted on double sticky tape, coated with a thin layer of gold, and observed using a Hitachi TM3000 scanning electron microscope (Hitachi, Tokyo, Japan). Analysis was conducted at an accelerating voltage of 15 kV, under a pressure of 100 Pa, and at a magnification of ×3000 [76].

### 3.11. Chemical Analysis

#### 3.11.1. Total Polyphenols Content (TPC)

The samples’ total phenolics content was evaluated through a spectrophotometric technique, involving a color reaction with Folin–Ciocalteau reagent [74]. The extracts underwent a double dilution with distilled water, and subsequent reactions were carried out in 96-well plates. A 5-fold diluted Folin–Ciocalteau reagent (40 µL) was added to 10 µL of the extact, followed by the addition of 250 µL of a 7% sodium carbonate solution after 3 min. Then, the solution was incubated for 60 min at room temperature in the absence of light exposure. The absorbance at 750 nm was measured utilizing a Multiskan Sky plate reader (Thermo Electron Co., Waltham, MA, USA). The absorbance of the blank sample, wherein the extract was substituted with the extraction reagent, was also recorded. Two repetitions were performed for each tested extract. For polyphenol content quantification, a calibration curve was established employing chlorogenic acid (Sigma Aldrich, Switzerland) within the concentration range of 0–100 g/mL. Findings are presented as milligrams of chlorogenic acid per 100 g of dry matter.

#### 3.11.2. Antioxidant Activity (AA)

To evaluate the antioxidant properties of the samples, spectrophotometric methods were employed. This involved assessing the capacity to reduce Fe3+ ions (RP), the 2,2-diphenyl-1-picrylhydrazyl radical (DPPH^•^), and the cation radical 2,2-azinobis(3-ethylbenzothiazoline-6-sulfonate) (ABTS^•+^) [77]. To induce the formation of free radicals, stock solutions of DPPH and ABTS were prepared 24 h prior to analysis. Initially, 25 mg of 2,2-diphenyl-1-picrylhydrazyl was weighed and transferred into a 100 mL volumetric flask, then diluted to 100 mL using a 99% methanol solution. The ABTS solution was created by dissolving 38.4 mg of 2,2-azinobis(3-ethylbenzothiazoline-6-sulfonate) in 10 mL of distilled water, followed by the addition of 6.6 mg of potassium persulfate. These solutions were then refrigerated. Prior to analysis, working solutions of the radicals were prepared by diluting the stock solutions with 80% ethanol. The dilution aimed to achieve a concentration displaying absorbance in a 1 cm cuvette at a wavelength of 515 nm for DPPH and 734 nm for ABTS, approximately reaching 0.7 AU (absorbance unit). The reactions were conducted in 96-well plates. The analyte solution was diluted fivefold. Initially, 10 µL of extract and 250 µL of radical solution were combined in the well, mixed, and the absorbance was measured for DPPH after 10 min at 515 nm, and for ABTS after 6 min at 734 nm, relative to 80% ethanol. Simultaneously, the absorbance of the radical working solutions was monitored. Antiradical activity was determined by the reduction in absorbance of the radical solution in the presence of an antioxidant, and it was expressed as mg of Trolox per gram of dried material. Each extract was analyzed in duplicate for this determination.

#### 3.11.3. Reducing Power (RP)

The analysis was conducted following the methodology outlined by Świeca [78], with minor adjustments. In a 96-well plate, 25 µL of the extract, 75 µL of distilled water, and 50 µL of 1% aqueous potassium ferric cyanide solution were combined. This mixture was then incubated in the dark at 50 °C using an incubator (INCU-Line ILS 10; VWR, Radnor, PA, USA). After 20 min, 50 µL of 10% trichloroacetic acid was added. Subsequently, 100 µL of the solution was transferred to an empty well, followed by the addition of 100 µL of distilled water and 20 µL of 0.1% iron (III) chloride solution. After 10 min, the absorbance values of the solutions were measured at 700 nm against the reagent sample using a plate reader. The iron ion reduction capacity for each sample was quantified as mg of Trolox. This determination was carried out in duplicate.

### 3.12. Statistical Analysis

The gathered data were statistically evaluated using Statistica 13.3 software (TIBCO Software Inc., Palo Alto, CA, USA). To assess the significance of differences in the average values of measured parameters of hydrogels, ANOVA was performed. Tukey’s test was used to determine the significance of the differences at α = 0.05. Furthermore, the results underwent evaluation via PCA and HCA.

## 4. Conclusions

Based on the findings of this study, it can be concluded that sequential thermal and ultrasound induction resulted in significant color changes and increased chroma values, reflecting less effective entrapment of the elderberry extract. Conversely, thermal induction followed by high hydrostatic pressure (HHP) treatment, particularly with extended durations (10 min), improved hydrogel strength, uniformity, and extract entrapment efficiency. Both treatments led to greater physical instability by altering the aggregate structures of the biopolymers. FT-IR analysis indicated that neither treatment caused chemical structural changes, although their effects on the hydrogel properties suggest physical interactions between the molecules. Thermal gravimetric analysis (TGA) revealed that both US and HHP treatments did not significantly impact the thermal degradation profile. Notably, HHP treatment significantly enhanced extract entrapment efficiency, resulting in better preservation of polyphenol content and antioxidant activity, attributed to the formation of a denser and more compact gel structure.

Overall, the hypothesis that combining thermal induction with high hydrostatic pressure (HHP) or ultrasound (US) treatments would enhance the functional properties and stability of pea protein–psyllium hydrogels for effective anthocyanin entrapment was partially verified. The sequential thermal and high hydrostatic pressure (HHP) induction outperformed the ultrasound-assisted induction in enhancing the structural integrity, entrapment efficiency, and antioxidant properties of elderberry extract-loaded pea protein–psyllium hydrogels. Despite concerns over physical stability, attributed to irregular distribution of pea protein–psyllium aggregates, HHP demonstrated significant promise. These findings underscore HHP as a promising method for developing functional matrices with enhanced retention of elderberry extracts. Further research is essential, particularly to enhance the entrapment efficiency, physical stability, and evaluate the suitability of sequential thermal and HHP induction for innovative food product development using elderberry extract-loaded pea protein–psyllium hydrogels.

## Figures and Tables

**Table 1:** Effects of sequential thermal and non-thermal induction on the color parameters (L*, a*, b*, C*, h, and ΔE) and extract entrapment efficiency (EE) of the obtained hydrogels.

Samples		Color Parameters					EE [%]
	L*	a*	b*	C*	h [°]	ΔE	
C	3.63 ^a^ ± 0.17	4.72 ^b^ ± 0.09	−0.37 ^b^ ± 0.06	4.7 ^b^ ± 0.1	274 ^b^ ± 1	-	19 ^c^ ± 0
U5	3.69 ^a^ ± 0.03	5.75 ^c^ ± 0.08	−0.10 ^c^ ± 0.06	5.8 ^c^ ± 0.1	271 ^a^ ± 0	1.1 ± 0.1	3 ^a^ ± 0
U10	3.66 ^a^ ± 0.03	5.89 ^c^ ± 0.04	−0.07 ^c^ ± 0.03	5.9 ^c^ ± 0.0	271 ^a^ ± 0	1.2 ± 0.0	9 ^b^ ± 1
P5	3.40 ^a^ ± 0.01	4.18 ^a^ ± 0.05	−0.74 ^a^ ± 0.04	4.3 ^a^ ± 0.0	280 ^c^ ± 0	0.7 ± 0.0	20 ^d^ ± 0
P10	3.44 ^a^ ± 0.14	4.19 ^a^ ± 0.09	−0.81 ^a^ ± 0.05	4.3 ^a^ ± 0.1	281 ^c^ ± 1	0.7 ± 0.1	33 ^e^ ± 0

The average (*n* = 3) values in columns with different letter symbols differ significantly (*p* < 0.05). The color difference between U5, U10, P5, and P10 in comparison to C was estimated using ΔE values: if 0 < ΔE < 1, the color is determined as not noticeable for the observer; 1 < ΔE < 2, only experienced observers can notice the difference in colors; 2 < ΔE < 3.5, unexperienced observers also notice the difference in colors; 3.5 < ΔE < 5, clear color difference in colors is noticed; 5 < ΔE, observer notices two different colors. Samples description: C—Control hydrogel induced via thermal induction; U5 and U10—Thermal induction followed by ultrasound treatment for 5 and 10 min, respectively; P5 and P10—Thermal induction followed by high hydrostatic pressure treatment for 5 and 10 min, respectively.

**Table 2:** Effects of sequential thermal and non-thermal induction on the instability index (physical stability) and textural and microrheological parameters of the obtained hydrogels.

Samples	Instability Index	Textural Parameters			Microrheological Parameters	
		Strength [N]	Adhesion [N]	Spreadability [N⋅s]	EI [nm^−2^]	SLB [-]
C	0.35 ^a^ ± 0.03	0.17 ^b^ ± 0.00	0.08 ^d^ ± 0.00	3.27 ^ab^ ± 0.76	0.057 ^c^ ± 0.005	0.01 ^a^ ± 0.00
U5	0.41 ^b^ ± 0.00	0.07 ^a^ ± 0.00	0.01 ^a^ ± 0.00	0.33 ^a^ ± 0.02	0.030 ^b^ ± 0.002	0.23 ^b^ ± 0.01
U10	0.46 ^c^ ± 0.00	0.07 ^a^ ± 0.00	0.01 ^a^ ± 0.00	0.19 ^a^ ± 0.00	0.017 ^a^ ± 0.003	0.28 ^c^ ± 0.01
P5	0.43 ^bc^ ± 0.01	0.19 ^c^ ± 0.00	0.04 ^c^ ± 0.00	4.71 ^b^ ± 0.91	0.016 ^a^ ± 0.003	0.33 ^d^ ± 0.01
P10	0.45 ^c^ ± 0.01	0.20 ^d^ ± 0.00	0.03 ^b^ ± 0.00	8.79 ^c^ ± 0.22	0.026 ^ab^ ± 0.001	0.35 ^e^ ± 0.01

The average (*n* = 3) values in columns with different letter symbols differ significantly (*p* < 0.05). Samples description: C—Control hydrogel induced via thermal induction; U5 and U10—Thermal induction followed by ultrasound treatment for 5 and 10 min, respectively; P5 and P10—Thermal induction followed by high hydrostatic pressure treatment for 5 and 10 min, respectively.

**Table 3:** Effects of sequential thermal and non-thermal induction on the total polyphenols content (TPC), antioxidant activity (expressed by ability to neutralize ABTS and DPPH radicals), and the reducing power of the obtained samples.

Samples	TPC [mg Chlorogenic Acid/100 g d.m.]	ABTS [mg TE/g d.m.]	DPPH [mg TE/g d.m.]	RP [mg TE/g d.m.]
C	18,403 ^b^ ± 221	4.8 ^b^ ± 0.1	6.8 ^b^ ± 0.0	0.87 ^b^ ± 0.03
U5	16,987 ^b^ ± 1668	3.6 ^a^ ± 0.3	8.8 ^c^ ± 0.8	0.98 ^c^ ± 0.01
U10	9317 ^a^ ± 1031	3.1 ^a^ ± 0.2	4.3 ^a^ ± 0.0	0.80 ^a^ ± 0.02
P5	18,865 ^b^ ± 278	4.9 ^b^ ± 0.2	7.7 ^bc^ ± 0.6	0.89 ^b^ ± 0.01
P10	22,741 ^c^ ± 1405	5.8 ^c^ ± 0.2	11.1 ^d^ ± 0.1	1.08 ^d^ ± 0.02

The average (*n* = 3) values in columns with different letter symbols differ significantly (*p* < 0.05). Samples description: C—Control hydrogel induced via thermal induction; U5 and U10—Thermal induction followed by ultrasound treatment for 5 and 10 min, respectively; P5 and P10—Thermal induction followed by high hydrostatic pressure treatment for 5 and 10 min, respectively.

**Table 4:** Explanation of hydrogel coding and the sequential inductions used.

Samples Code	Primary Induction	Secondary Induction	Secondary Induction Duration [min]
C	Heating at 80 °C for 30 min	-	-
U5		Ultrasound treatment (25 kHz, 70 W, 100% pulse, 100% amplitude)	5
U10			10
P5		High hydrostatic pressure (500 MPa)	5
P10			10

## Data Availability

The data used in this contribution are available upon request.
